# Dr. James McClintock

**Published:** 1857-07

**Authors:** 


*Dr. James McClintock.*—One of the most painful events which we have ever been called upon to record, has recently occurred in Philadelphia. Dr. James McClintock, formerly a well-known teacher, a few years since sold his name to Thomas McElrath, of the New York Tribune, and Burton, of Burton's Theatre, to be used *ad captandum vulgus* in the sale of a long list of nostrums, of which McClintock furnished the recipes and got $5000 therefor. The speculation failed. The philanthropist of the Tribune and the low comedian of the theatre, sunk $70,000 in the operation, and acquired a valuable experience in quackery. McClintock was left upon his oars, and finding himself poor, with a damaged reputation, he plays the "repentant sinner," and gets the appointment of Resident Physician-in-Chief, at Blockley Hospital, Philadelphia, a post of honor and profit. To keep up the cant of returning to the fold and making atonement for his errors, he publishes the recipes of his quack remedies in that very appropriate receptacle, the Philadelphia Medical Journal. He sold these recipes once for $5000, and it would seem to the ordinarily honest man that this latter proceeding betrays Messrs. McElrath and Burton, as badly as the sale itself betrayed McClintock's honor.

The Assistant Physicians at Blockley, have all resigned—to their honor be it spoken. They have taken a manly course in view of this insult to the profession, this offer of a reward to scapegraces.

